# The Effects of Orexin Receptor Antagonism During Early Withdrawal in Stimulant Use Disorder: Protocol for a Proof-of-Concept Study

**DOI:** 10.2196/83842

**Published:** 2026-04-08

**Authors:** Heather Webber, Nikky Tran, Rita Blake, Sarah Goldman, Trevor George, Bill Bailey, Robert Suchting, Jin Yoon, Jessica Vincent, Michael Weaver, Joy Schmitz, Scott Lane

**Affiliations:** 1 Faillace Department of Psychiatry and Behavioral Sciences, McGovern Medical School, University of Texas Health Science Center at Houston, 1941 East Rd, BBSB 1st floor, Houston, TX, 77054, United States, 1 7134862723; 2 The Right Step - Houston, Houston, TX, United States; 3 Cenikor Foundation, Houston, TX, United States

**Keywords:** stimulants, substance use disorder, suvorexant, treatment, orexin, sleep, stress, reward

## Abstract

**Background:**

Stimulant use disorders (StimUDs), including cocaine and methamphetamine use disorders, remain a major public health concern in the United States with no US Food and Drug Administration (FDA)–approved pharmacological treatments. The early recovery period following stimulant use is marked by sleep disturbances, heightened stress reactivity, and dysregulated reward processing, contributing to high rates of resumption of use. Evidence from animal and human studies supports the orexin neuropeptide system as a promising therapeutic target for minimizing these disruptive mechanisms. Suvorexant, an FDA-approved dual orexin receptor antagonist for insomnia, has previously been shown to improve sleep, reduce stress, and attenuate craving in the first-in-human study with non–treatment-seeking individuals with cocaine use disorder.

**Objective:**

The primary objective of this randomized open-label clinical trial is to evaluate the effects of suvorexant on sleep, stress, reward processing, and craving during early abstinence in treatment-seeking individuals with StimUD. This proof-of-concept trial represents the first mechanistic evaluation of suvorexant in a residential sample of individuals with StimUD.

**Methods:**

A total of 40 participants, recruited from a residential treatment facility, will be randomized (1:1) to either 7 days of nightly suvorexant (10 mg) or treatment as usual. Participants’ sleep will be monitored using an activity tracker watch, and they will complete a sleep diary each night to confirm bedtimes and wake-up times. Participants in the suvorexant group will receive the study medication for 7 nights. On premedication and postmedication study days, participants will be transported to the Center for Neurobehavioral Research on Addiction to complete the study sessions. At both study sessions, participants will complete self-report and safety measures (safety measures for the suvorexant group only) followed by administration of a stress reactivity test and an electroencephalographic assessment.

**Results:**

The study was funded in January 2025 and is currently ongoing. We began enrollment of the first participant prior to funding in July 2024. At the time of manuscript submission (September 2025), a total of 8 participants had been enrolled in the study toward the planned enrollment of 40 participants. This study is expected to conclude in January 2027.

**Conclusions:**

Findings from this study will provide initial evidence for the mechanisms involved in suvorexant for StimUD, with the long-term goal of repurposing orexin-based therapy to regulate neurobehavioral mechanisms and inform future medication development for StimUD.

## Introduction

Stimulant use disorders (StimUDs), including cocaine and methamphetamine use disorders, represent a growing public health crisis in the United States. Unlike opioid use disorder, for which multiple pharmacotherapies exist, StimUDs remain without a US Food and Drug Administration (FDA)–approved medication, posing a significant challenge for clinical management. Historically, pharmacological interventions have targeted monoaminergic systems—particularly dopaminergic pathways—with limited efficacy [1]. This therapeutic gap underscores the urgent need for novel, mechanism-based treatments that address the neurobiological underpinnings of stimulant addiction and withdrawal.

One promising target is the orexin (hypocretin) system, a neuropeptide network originating in the lateral hypothalamus that plays a central role in regulating arousal, stress, and reward processing. Converging evidence from preclinical and translational studies implicates orexin signaling in the maintenance of drug-seeking behavior, particularly under conditions of heightened stress or disrupted sleep [2]. Of particular relevance to early abstinence is the role of orexin in sleep-wake regulation. Sleep disturbances are a hallmark of stimulant withdrawal and are strongly predictive of resumption of use [3]. Therefore, pharmacological modulation of orexin receptors may offer a dual mechanism of action—ameliorating sleep disruption while attenuating drug-motivated behavior.

Dual orexin receptor antagonists, such as suvorexant, have demonstrated efficacy in improving sleep architecture and reducing hyperarousal in individuals with insomnia. In a recent proof-of-concept study, suvorexant was shown to improve sleep quality, reduce stress, and attenuate craving in non–treatment-seeking individuals with cocaine use disorder [4]. Building on these findings, this study aims to evaluate the effects of suvorexant during early withdrawal in treatment-seeking individuals with StimUD, including those using both cocaine and methamphetamine, as they transition into residential addiction treatment.

To extend the mechanistic understanding of suvorexant’s effects, we incorporate electroencephalography (EEG) measures of brain function. Two neural markers are of particular interest. First, resting-state alpha power (8‐13 Hz), typically observed over posterior scalp regions during relaxed wakefulness, is sensitive to sleep pressure and may serve as an objective index of daytime neural deactivation [5]. Preliminary data from our group suggest that increased alpha power is associated with poorer subjective sleep quality in people who use stimulants. Second, the reward positivity (RewP) component—an event-related potential elicited by positive prediction errors—indexes reward sensitivity and is often blunted in individuals with substance use disorders [6]. Given the orexin system’s role in (1) modulating mesolimbic dopamine pathways and (2) normalizing sleep architecture, suvorexant may enhance neural responsiveness to nondrug stimuli (potentially both cues and rewards), thereby supporting recovery-related motivational processes.

The primary objective of this translational study is to evaluate the effects of suvorexant on sleep during early abstinence from stimulants—a critical window in which effective withdrawal management may reduce the risk of return to stimulant use. The secondary objective is to examine whether suvorexant will also improve reward processing and reduce stress and craving during early abstinence. By integrating behavioral, subjective, and neurophysiological measures, this investigation represents the first mechanism-focused clinical evaluation of orexin antagonism in StimUD. We hypothesize that suvorexant, relative to treatment as usual (TAU), will improve sleep outcomes during early abstinence (aim 1). We also hypothesize that the secondary outcomes (EEG resting alpha power, acute stress reactivity, non–drug-reward processing, and drug demand) will improve from pretreatment to posttreatment in the suvorexant group compared with the TAU group (aim 2). Finally, we will examine treatment engagement and the effects of the treatment group (suvorexant vs TAU) on posttreatment (days 13‐30) residential program length of stay and completion rates (aim 3). We hypothesize that participants in the suvorexant group, compared with the TAU group, will demonstrate longer posttreatment lengths of stay and higher residential program completion rates.

## Methods

### Study Design

**Figure 1.**
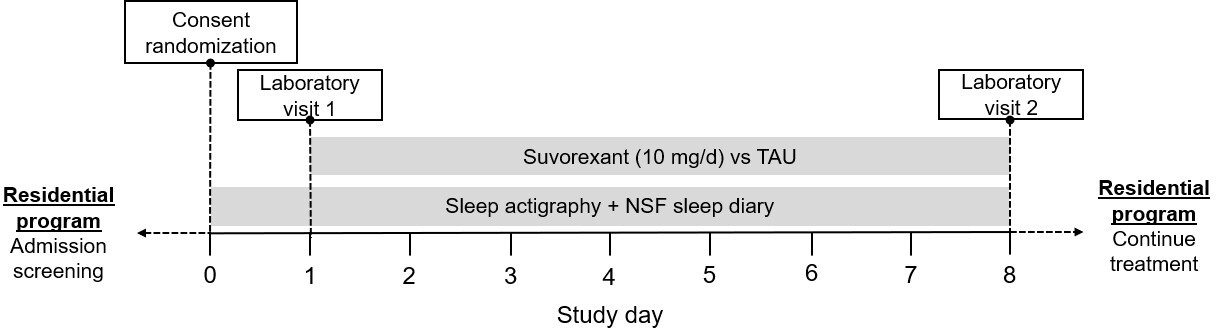
Study flow diagram. Consent and randomization will occur virtually. Laboratory visits will occur at the Center for Neurobehavioral Research on Addiction. All other activities will occur at the inpatient facilities. NSF: National Sleep Foundation; TAU: treatment as usual.

We will use an open-label, randomized 2-group design. Participants (n=40) will be randomized to receive 7 days of suvorexant (10 mg) or TAU, consisting of supportive care and symptomatic medication. Figure 1 outlines the schedule of events. Informed consent, eligibility criteria, and baseline sleep data (via actigraphy) will be assessed on day 0 of the study via telephone or video call. Day 0 will ideally occur around 4 days (but within the first week) following admission to the residential program. On day 1 and day 8, participants will be transported to the outpatient treatment research clinic at the Center for Neurobehavioral Research on Addiction (CNRA) to complete the study sessions. At each study session, participants will complete self-report and safety measures (safety measures for the suvorexant group only) followed by administration of the stress reactivity test and then the EEG assessment. Details regarding each of these procedures are provided in subsequent sections. Participants in the suvorexant group will begin receiving the study medication, administered by residential nurses, from day 1 up until day 8 (ie, nights 1‐7). All participants will remain in the residential program until either regular discharge at approximately 30 days (“successful completion”) or early departure (eg, patient-directed discharge or leaving against medical advice). This design captures the critical first 2 weeks of stimulant withdrawal, when sleep disruptions and other withdrawal-related symptoms are typically most pronounced [7].

### Recruitment

Participants will be recruited directly from 2 local inpatient residential treatment facilities in the greater Houston area (The Right Step Houston and Cenikor), both of which provide similar therapeutic programming and services. Patients meeting the preliminary eligibility criteria will be referred to the CNRA by the residential programs. Additional recruitment will include online advertisements and outreach through the University of Texas Health Science Center at Houston (UTHealth Houston), Department of Psychiatry Recruitment Registry. Individuals recruited from outside the residential programs must voluntarily seek admission to one of the residential facilities before participating. Eligibility will be assessed using residential intake records (including a history and physical or psychological assessment) and a phone-based screening. Eligible candidates will participate in a phone interview including the consent process and administration of the structured clinical interview for *Diagnostic and Statistical Manual of Mental Disorders, Fifth Edition* (*DSM-5*) [8] by trained CNRA staff (ie, licensed professional counselor, doctoral student, or postdoctoral fellow). The principal investigator (PI; author HW) and the medical director (author MW) will review intake and phone-interview information to determine final participant eligibility.

### Participants

Participants will be eligible for the study if they are aged between 18 and 65 years and meet the *DSM-5* criteria for a primary StimUD, specifically related to cocaine or methamphetamine. All participants must be fluent in English and capable of fully understanding and providing informed consent for study procedures. These inclusion criteria ensure that participants have the condition of interest and can engage meaningfully with study requirements.

Participants will be excluded if they meet any of the following criteria: (1) diagnosis of an opioid use disorder of any severity; (2) diagnosis of greater than moderate substance use disorder for any substance other than cocaine or methamphetamine; (3) currently receiving medication treatment for withdrawal from any substance; (4) presence of medical conditions that contraindicate the use of suvorexant (eg, severe pulmonary disease, severe cardiovascular disease or clinically abnormal electrocardiogram, severe liver or kidney disease, or sleep disorder—particularly narcolepsy); (5) use of medications known to interact with suvorexant (eg, monoamine oxidase inhibitors, anticonvulsants, haloperidol, phenothiazines, anesthetics, and any sedative); (6) pregnancy or breastfeeding; (7) BMI >30 kg/m^2^ (women only); (8) current *DSM-5* psychiatric disorder or neurological disease requiring ongoing treatment that would make participation unsafe; (9) history of seizure disorder; and (10) history of head injury with loss of consciousness within the past 5 years.

### Randomization

Participants will be randomly assigned in a 1:1 allocation ratio to each treatment group using block randomization. Allocation will use permuted blocks with randomly varying per-arm sizes to minimize predictability while preserving balance. The randomization sequence will be generated in the R Statistical Computing Environment (R Foundation for Statistical Computing) via the blockrand package [9]. A blinded code will be provided to an unblinded study team member who will execute the script, export the allocation file, and upload it to the project’s randomization module in REDCap (Research Electronic Data Capture). The full table will be access restricted such that staff members will see only the next assignment when needed. A read-only copy will be kept safe on a secure drive for emergency unblinding.

### Intervention

Suvorexant is a dual orexin receptor antagonist approved by the FDA for the treatment of insomnia. In this study, we will administer 10 mg of suvorexant for 7 nights. Two prior studies from our group suggested that suvorexant could be safely administered to individuals with cocaine use disorder [4] and methamphetamine use disorder [10]. The half-life is about 12 hours on average, and steady-state plasma concentrations occur in 3 days with daily administration [11]. A nurse at the residential programs will administer the medication to participants in the suvorexant group each evening before bedtime, at the same time nightly (around 10 PM), with a medication log to monitor compliance.

All participants will receive TAU, consisting of standard programming from the inpatient facilities, including a combination of individual therapy, group therapy, 12-step meetings, case management, and recreational therapy. Participants in the suvorexant group will receive suvorexant and will be asked to avoid medications listed in the exclusion criteria, including sleep-related medications. Participants in the TAU group will continue their usual medication regimen as prescribed by the inpatient facility staff. A comprehensive list of medications prescribed for each patient will be collected. If possible, statistical analyses will attempt to control for other medication exposure.

### Sleep Assessment

Sleep will be monitored using an activity tracker watch (wGT3X-BT; ActiGraph, LLC) from night 0 to the morning of day 8. Data will be downloaded via the ActiLife software onto a UTHealth Houston computer and securely stored on IT-protected servers. Outcome measures derived from the actigraphy data will include total sleep duration and wake after sleep onset, two widely recognized indicators of sleep quality [12]. To supplement the actigraphy data, participants will complete the National Sleep Foundation Sleep Diary each night to confirm bedtimes and wake-up times for quality control purposes. Additional self-report sleep measures will be collected as described in subsequent sections.

### Study Visit Measures

#### Self-Report and Behavioral Assessments

##### Sleep

The Pittsburgh Sleep Quality Index total summary score will be used to assess baseline sleep quality [13-15]. We will use the Insomnia Severity Index (ISI) [16] and Patient-Reported Outcomes Measurement Information System–Short Form (PROMIS-SF; Sleep Disturbance and Sleep-Related Impairment items) [17] as the main outcome variables. ISI and PROMIS-SF will be assessed on day 5 and day 12 at the laboratory visits to capture changes in sleep over the past 7 days.

##### Stress

Subjective stress levels will be assessed with the Depression, Anxiety, and Stress Scale-21 Items (DASS-21), a validated and reliable measure of core symptoms associated with depression, anxiety, and stress [18]. We will also use a visual analog scale (VAS; “Please rate your current stress level”: 0=no stress, 10=extreme stress) to assess current subjective stress levels [19].

##### Craving and Demand

The Stimulant Craving Questionnaire (STCQ) will be used to assess current craving [20]. The STCQ is a 10-item inventory that is useful in measuring craving across both stimulant types (cocaine and methamphetamine). The drug purchasing task will be used to assess demand for stimulants. During the hypothetical drug purchasing task, participants indicate how much of the drug they would use based on increasing prices [21-25]. Participants will complete a version of the drug purchasing task based on their primary stimulant use disorder (cocaine or methamphetamine).

##### Safety, Side Effects, and Other

A 31-item questionnaire will be administered to participants in the suvorexant group only to assess side effects and adverse events. The Columbia Suicide Severity Rating Scale [26] will be administered at each laboratory visit, and the Beck Depression Inventory [27] will also be included in the self-report battery. Additionally, at the first in-person visit, the Addiction Severity Index will also be administered to assess substance use severity and collect relevant demographic and addiction-related information [28].

### Laboratory Assessments

#### Sleep

Resting-state EEG will be collected on laboratory days to assess the waking effects of sleep quality. The outcome variable will be alpha power (8‐12 Hz), which reflects a state of wakeful rest [5,29]. Both eyes open and eyes closed conditions will be measured and analyzed separately. Resting alpha power will be collected at each laboratory visit day. Refer to the EEG Protocol section for details.

#### Stress

The socially evaluated cold pressor test will be used to measure stress reactivity. This task reliably activates the sympathetic nervous system and the hypothalamic-pituitary-adrenal (HPA) axis, as evidenced by increases in heart rate (HR), blood pressure (BP), and cortisol levels [30-33]. The social evaluative component is used to enhance HPA-axis activation. Participants will face a computer monitor showing their face using a webcam and a confederate wearing a white laboratory coat and clipboard will also be in the room. Participants will be informed that the camera is recording their facial reactions for later analysis and that the physician in the white laboratory coat will also be taking notes on their stress responses. No facial recordings or actual notes will be taken. During the cold pressor test (CPT), participants will submerge their arm in an ice-water bath. Physiological measures of stress reactivity will include HR, BP, and salivary cortisol, consistent with previous studies from our group [19,34]. Saliva samples will be collected before and after the CPT using the Cortisol-Salivette system (Sarstedt) and analyzed with the Cortisol ELISA Kit (Enzo Life Sciences), per manufacturer instructions. Subjective stress will be assessed before and after the CPT using a single-item VAS question, and HR and BP will also be recorded before, during, and after the CPT.

#### Reward Processing

EEG will also be recorded during the doors task to elicit the RewP [6]. In this task, participants are shown 2 doors, one of which has a hypothetical prize behind it. Participants guess which door hides the prize and then receive feedback indicating the outcome (up arrow=monetary win, down arrow=monetary loss). The RewP will be quantified as the mean amplitude in response to monetary feedback. Refer to the EEG Protocol section for details.

### EEG Protocol

On laboratory days, participants will be seated comfortably in front of a monitor, fitted with the EEG cap, and the electrodes will be prepared with conductive gel. EEG will be collected using a 64-channel actiCAP electrode cap, amplified with BrainAmp MR (Brain Products GmbH) and digitized using BrainVision Recorder (Brain Products GmbH). The sampling rate will be 250 Hz, and data will be filtered with a 0.1 Hz high-pass filter and a 100 Hz low-pass filter.

Resting-state EEG will be collected prior to the doors task. We will collect resting-state EEG for 3 minutes with eyes open and eyes closed.

EEG data reduction will be performed with Brain Vision Analyzer 2 (Brain Products GmbH), MATLAB (MathWorks), and BESA (BESA GmbH). Resting-state data will be analyzed using the fast Fourier transform. The outcome of interest will be alpha power (8‐12 Hz). The doors task data will be segmented (from 200 ms prestimulus to 800 ms poststimulus) by the following conditions: wins and losses. The RewP will be defined as the mean poststimulus amplitude over the frontal electrodes.

### Dissemination Plan

The PI will be responsible for keeping the study record up to date on a yearly basis and for posting the results of the clinical trial on ClinicalTrials.gov. Findings will also be disseminated through conference presentations and publications in peer-reviewed scientific journals.

### Statistical Analysis Plan

Analyses will be conducted within a generalized linear mixed modeling framework, which provides the flexibility to accommodate non-Gaussian outcomes via link functions and repeated or multilevel measurements via random effects. Traditional methods (eg, both 1-tailed and 2-tailed *t* tests and ANOVA) are subsumed as special cases, and the framework supports further extensions for nonlinear effects, time-varying predictors, and multivariate outcomes. For aims 1 (sleep) and 2 (all secondary measures), analyses will model each outcome variable as a function of the time and treatment interaction, controlling for main effects (fixed effects: outcome=β_0_ + β_1_ × treatment *+* β_2_ × time *+* β_3_ × treatment × time), with random effects to handle correlated observations. For aim 3 (treatment completion), analyses will model each outcome as a function of treatment.

All models will use distribution families and link functions as appropriate for each outcome (eg, dichotomous outcomes: binomial, with logit link). Longitudinal models evaluating change via interaction terms (eg, treatment × time) will be reduced to main effects if change is unsupported, and both models will be reported. Model fit information criteria (Akaike information criterion; leave-one-out information criterion) will be used to select structural components including multilevel (eg, random intercepts and slopes) and nonlinear terms. Analyses will be performed in R [35] via packages lme4 [36], rstan [37], and brms [38]. Potential confounders will be identified empirically [39,40] (ie, associated with both predictor and outcome) and modeled with and without adjustment; models with unchanged inference will favor parsimony. Sample characteristics (eg, sex, age, and education) will be evaluated as potential confounders. Analyses will be run in parallel using frequentist and Bayesian models. Frequentist results yield the probability of the data (or data more extreme) given the null hypothesis, whereas Bayesian results directly yield the probability of an alternative hypothesis [41,42]. Frequentist models will use *α*=.05 with false discovery rate correction for exploratory tests. Bayesian priors will be weakly informative (β*~* N(0,1); sd~ Half-Cauchy(0,1); correlation matrices ~ LKJ(2)). Bayesian models will be evaluated via posterior probability (PP) threshold guidelines in the literature [43,44], suggesting that PP=75% to 90% indicates moderate evidence, PP=91% to 96% indicates strong evidence, and PP=97% or above indicates very strong to extreme evidence. Posterior predictive checking, scale reduction factors (“rhat”), and effective sample size will be used to evaluate Bayesian assumptions. Evaluation for frequentist models will use graphical evidence and statistical tests. Generalized linear mixed models inherently accommodate outcome values missing at random; other missing values will be handled by imputation or pattern-mixture modeling. Analyses will follow intention-to-treat principles.

The current proof-of-concept study is designed primarily to estimate a plausible range of treatment effects rather than to provide definitive hypothesis tests. To characterize expected inferential performance at the proposed sample size (n=40; 20 per arm), Monte Carlo simulation–based design analyses were conducted (1000 iterations). Data were generated under a linear mixed model with a treatment × time interaction, AR(1) correlated repeated measures (ρ=0.50), and the weakly informative priors specified earlier. At this sample size, assuming 10% attrition (n=36), simulations demonstrate at least an 80% probability (analogous to statistical power) of achieving moderate evidence (PP ≥75%) for standardized average treatment effects, unfolding in linear fashion over time, as small as Cohen *d*=0.26, and 80% probability of strong evidence (PP ≥91%) at Cohen *d*=0.39. Wide credible intervals are anticipated at this sample size, and results will be interpreted accordingly as preliminary effect size estimates to inform future definitive trials.

### Ethical Considerations

The study protocol was approved by the Committee for the Protection of Human Subjects at UTHealth, Houston, Texas (HSC-MS-24‐0330). Consent will be obtained virtually at the inpatient facility by a member of the CNRA team via voice or video call. Interested participants will e-sign the consent via REDCap. A copy of the signed consent can be printed or emailed to the participant. The PI will be responsible for ensuring compliance with the policies of the local institutional review board (the UTHealth Houston Committee for the Protection of Human Subjects). All participant data will be kept confidential. Data and safety oversight will include staff training, weekly audits of data collection and entry, medical screening with results reviewed by an on-site nurse or physician, use of standardized assessments, continued medical monitoring during the study, and procedures to monitor medication compliance. A data safety monitoring board will provide additional, independent oversight of data related to participant safety. All adverse events will be recorded for duration, severity, and frequency, and an evaluation will be made as to the event’s relationship to the study drug, classified as unrelated, unlikely, possible, or probable. Participants will be compensated for their time as follows: US $5 for the screening and consent, US $40 for laboratory visits, US $10 for questionnaires, US $1 per day for medication adherence, and US $50 completion bonus.

## Results

The study was funded in January 2025 and is currently ongoing. We began enrollment of the first participant prior to funding in July 2024. As of the submission of the manuscript (September 2025), a total of 8 participants had been enrolled in the study toward the planned enrollment of 40 participants. This study is expected to conclude in January 2027. No outcome data are currently available.

## Discussion

### Summary

Due to its mechanism of action, suvorexant may aid in preventing resumption of use in patients recently abstinent from stimulants via improvements in sleep, stress, and reward processing. The current open-label, randomized clinical trial will assess this hypothesis by randomizing patients with StimUD to receive either suvorexant or TAU for 7 nights at the local residential treatment facilities. We anticipate participants will demonstrate good adherence to suvorexant, and we will observe these improvements in both subjective and objective measures compared with the TAU group. We also anticipate that these mechanisms will be associated with increased treatment engagement demonstrating longer posttreatment lengths of stay and higher residential program completion rates.

Orexin receptor antagonism has shown promise in reducing stimulant-seeking behavior in preclinical studies, but there has been limited research in humans. This study builds upon the human literature that has examined the effects of suvorexant in StimUD, which has yielded mixed findings. Previously, in the preliminary proof-of-concept study, we found initial human evidence that dual orexin receptor antagonism with suvorexant improved multiple mechanistic processes in cocaine use disorder [4]. In contrast, in an acute cocaine self-administration study, Stoops et al [45] found that suvorexant increased the participants’ motivation for cocaine rather than decreasing it. While the methods varied between these studies and could account for the differences in results, this study will add to the growing literature in the area.

There are several strengths and seminal innovations to the current proof-of-concept trial design, but it is not without limitations. This will be the first study to examine the effects of suvorexant during early withdrawal from stimulant use (both cocaine and methamphetamine) in treatment-seeking patients entering residential treatment for StimUD. Furthermore, the well-validated actigraphy-focused sleep assessment will contribute to our understanding of the mechanistic effects of suvorexant in this population. Another first will be leveraging EEG to assess the effects of suvorexant on the waking brain through both resting-state alpha power and the RewP. Finally, this trial will potentially provide effect size estimates of the efficacy of suvorexant regarding the treatment of StimUD, by assessing inpatient treatment completion rates. Despite these strengths, this study will be limited by the open-label design. This could result in the subjective outcomes (eg, sleep quality ratings, stress ratings, and craving scores) being vulnerable to placebo effects, expectancy bias, and demand characteristics. These outcomes will be interpreted cautiously in the statistical analysis. To overcome these concerns, we have included several validated objective outcomes (eg, actigraphy, EEG, and physiological measures), which are less susceptible to participant expectations and will be prioritized when drawing conclusions about treatment effects. Findings from this study will be exploratory and future blinded, placebo-controlled trial designs will establish definitive efficacy.

### Conclusions and Future Directions

Notably, this trial was designed as a proof-of-concept study primarily concerned with deriving a preliminary range of plausible effect sizes for mechanistic effects and the effect on treatment completion. The study sample size was selected based on available funding and time to complete the study tasks. Therefore, the results of this study are meant to guide future fully powered trials rather than provide accurate efficacy estimates. A further limitation of this study is the lack of a placebo control and the open-label design. This design introduces potential biases into subjective measures due to participants’ knowledge that they are receiving the treatment. As replication in a larger controlled trial is still needed, a negative finding would not rule out efficacy. Positive findings will also need to be confirmed in a larger sample. A TAU control condition was selected as the next best option and will provide information regarding how add-on suvorexant compares to supportive care and symptomatic medication alone. Critically, the use of several objective measures of main outcomes (actigraphy, HR, BP, cortisol, and EEG) will partially offset the influence of the open-label medication. This translational research approach will be the first mechanism-focused clinical investigation of suvorexant for the treatment of StimUD. FDA approval of suvorexant for the treatment of primary insomnia helps pave the way for rapidly advancing the repurposing of orexin-based therapies for drug addiction treatment.

## Abbreviations

BP: blood pressure
CNRA: Center for Neurobehavioral Research on Addiction
CPT: cold pressor test
DASS-21: Depression, Anxiety, and Stress Scale-21 Items
*DSM-5*: *Diagnostic and Statistical Manual of Mental Disorders, Fifth Edition*
EEG: electroencephalography
FDA: Food and Drug Administration
HPA: hypothalamic-pituitary-adrenal
HR: heart rate
ISI: Insomnia Severity Index
PI: principal investigator
PP: posterior probability
PROMIS-SF: Patient-Reported Outcomes Measurement Information System–Short Form
REDCap: Research Electronic Data Capture
RewP: reward positivity
STCQ: Stimulant Craving Questionnaire
StimUD: stimulant use disorder
TAU: treatment as usual
UTHealth Houston: University of Texas Health Science Center at Houston
VAS: visual analog scale
